# Investigation of Cytotoxic and Antimicrobial Effects of Polyanhydride-Based Poly[(maleic anhydride)-*co*-(vinyl Acetate)] Conjugates Combined with Methotrexate and Gemcitabine in Breast Cancer Treatment

**DOI:** 10.1021/acsomega.4c10445

**Published:** 2025-03-22

**Authors:** Tutku Tunç, Gülderen Karakuş, Zeynep Sümer

**Affiliations:** † Department of Pharmaceutical Microbiology, Sivas Cumhuriyet University Faculty of Pharmacy Sivas, 58140 Sivas, Türkiye; ‡ Department of Pharmaceutical Basic Sciences, Sivas Cumhuriyet University Faculty of Pharmacy Sivas, 58140 Sivas, Türkiye; § Department of Medical Microbiology, Sivas Cumhuriyet University Faculty of Medicine Sivas, 58140 Sivas, Türkiye

## Abstract

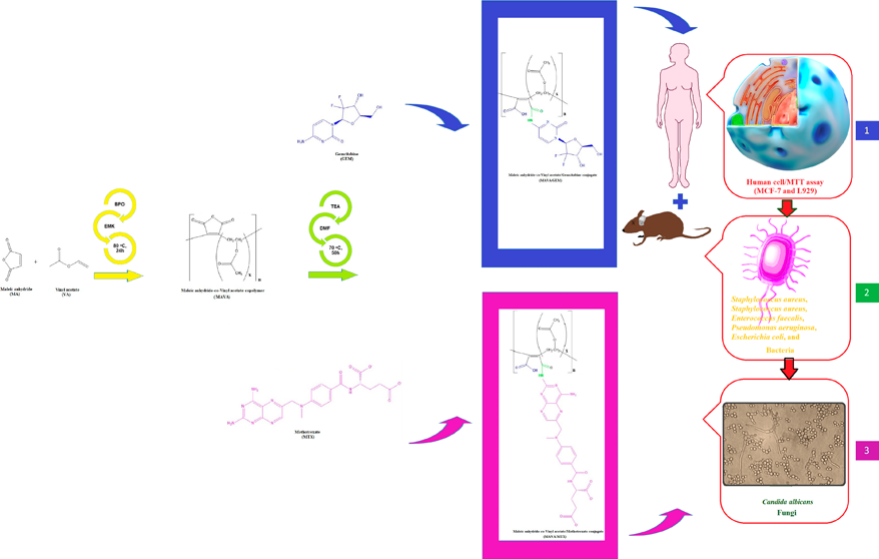

Studies aimed at increasing the efficacy of chemotherapeutic drugs and reducing or completely eliminating their side effects are frequently encountered. In our study, we considered methotrexate (MTX), which is in the category of anticarcinogenic and anti-inflammatory drugs, and gemcitabine (GEM), which is used in the treatment of breast, testicular, ovarian, etc. cancers. GEM, which is used in the treatment of breast, testicular, ovarian, etc. cancers, was covalently bonded to maleic anhydride vinyl acetate (MAVA) copolymer, and new polymer–drug conjugates (MAVA–MTX and MAVA–GEM) were obtained to reduce or eliminate the side effects of these drugs and to investigate the cytotoxic and antimicrobial effects of the new conjugates. The conjugation reaction was carried out in the presence of a triethylamine catalyst in dimethylformamide medium at 70 °C. Chemical structure elucidation of the copolymer (MAVA) and conjugates (MAVA–MTX and MAVA–GEM) was carried out by Fourier transform infrared (FTIR) and Nuclear Magnetic Resonance (^1^H NMR) spectroscopy. Anticancer activity was determined by the MTT assay in MCF-7 (breast cancer), and L929 (mouse fibroblast) cell lines. The synthesized copolymer and conjugate structures were proved by FTIR and ^1^H NMR spectra. It was determined that the conjugates did not form an inhibition zone on the test microorganisms. MIC values were found to be moderately effective compared with reference sources. The anticancer activities of MAVA–MTX and MAVA–GEM conjugates were significantly higher than those of methotrexate and GEM. The higher anticancer activity of the synthesized MAVA–MTX and MAVA–GEM conjugates compared with the drug they contain suggests that they can be a potential drug candidate in the treatment of breast cancer. In addition, the conjugates showed less toxic effect on a healthy L929 cell line at 6 different concentrations compared to free drugs. This can be shown as a significant improvement in reducing one of the most important side effects of the drug, such as toxicity.

## Introduction

Cancer is a chronic disease that causes massive mortality worldwide and cancer cases are constantly increasing.^1^ Worldwide, cancer is the second leading cause of death after cardiovascular disease.^2^ Although there are many types of cancer, the most common are breast, colorectal, and lung cancer. While breast cancer is the most common cancer in women, lung cancer is the most common cancer in men.^3^

Cancer treatment includes radiotherapy, surgery, hormonal therapy, immunotherapy, and chemotherapy (anticancer drugs).^1^ It is also possible to treat cancer with combination therapy, which involves the use of two or more anticancer agents. In addition to combination therapy, a polymer-based drug delivery system is another potential strategy reported to enhance the therapeutic efficacy of anticancer agents. The treatment method applied depends on the location and stage of the tumor. The use of anticancer drugs is the most commonly used method and they are therapeutic agents that can be used to target proteins, tissue environment, and genes responsible for cancer growth.^4^

There are many factors that limit the effectiveness of the drugs used in chemotherapy. These include drug resistance, toxicity, factors related to tumor–drug interaction, factors related to drug pharmacokinetics and pharmacology, and patient-related factors.^5^ The clinical use of many chemotherapy drugs is limited due to their toxic side effects and low therapeutic index.^6^ For this reason, various proposals have been developed to ensure that the drugs are localized where they need to go in the body and to reduce side effects. Polymer-based drug delivery systems are used in biomedical applications to deliver therapeutic agents to the target biological environment. They exhibit different properties such as reduced drug toxicity, improved patient compliance, increased drug solubility, improved drug bioavailability, biocompatibility, and biodegradability, control of the drug release mechanism, and maintaining drug activity during circulation.^4,7^

Researchers are creating new pharmacological systems, particularly using polymers, as a result of the severe side effects of the medications used to treat breast cancer and their lack of efficacy in treating the tumor. Gemcitabine, a drug used to treat breast cancer and commonly found in polymer–drug conjugate investigations, has a short half-life and a very toxic effect on tissues, which restricts its therapeutic use.^8,9^ Leukemia, lymphoma, solid tumors, psoriasis, and rheumatoid arthritis are all frequently treated with methotrexate (MTX), an antimetabolite with antineoplastic properties. It is a chemotherapeutic medication that prevents the synthesis of DNA and RNA.^10^

The IUPAC name of methotrexate is (2*S*)-2-[[4-[(2,4-diaminopteridin-6-yl)methyl–methylamino]benzoyl]amino]pentanedioate.^11^

The IUPAC name of gemcitabine is 4-amino-1-[(2*R*,4*R*,5*R*)-3,3-difluoro-4-hydroxy-5-(hydroxymethyl)oxolan-2-yl]pyrimidin-2-one.^12^

Antimicrobial agents have been developed to stop the growth of or kill microorganisms. Antimicrobial polymers have been the subject of intense academic research over the past few years.^13,14^ Polymers classified as antimicrobial either exhibit their antimicrobial activity or are conjugated to other antimicrobial compounds such as antibiotics.^15^ Antimicrobial activity is tightly related to various factors such as molecular weight, type of counterion, length of the spacer between the polymer and the active site, or hydrophilic–hydrophobic balance. The use of antimicrobial polymers has many advantages compared to conventional solutions. First of all, they are materials with long-term activity; second, they are nontoxic, chemically stable, and nonvolatile; third, they can act selectively. The great application potential of antimicrobial polymers is utilized in the food, medical, and textile industries. Maleic anhydride (MA)-based polymers are one of the examples of such materials.^16^

Candidate polymers as drug conjugates should meet certain criteria such as biocompatibility and have suitable functional groups such as –COOH, –NH_2_, –OH, etc. for covalent conjugation with drugs, water solubility, low PDI value, and easy availability. MA copolymers are notable for their unique properties such as biocompatibility, low toxicity^17^ easy availability, and ability to modify hydrophilicity and hydrophobicity depending on comonomer choice and water solubility in pharmaceutical and medical applications.^18^ Therefore, it is possible to modify and improve the properties and uses of this class of polymers. Drugs can be easily attached to MA copolymers through the anhydride cycle or comonomer reactive groups. MA copolymers can be used as drugs, as supports for bioactive molecules, or as conjugates in drug-controlled release systems. One of the main purposes of using polymer–drug conjugates is to improve the properties of the selected drug in terms of stability, activity, bioavailability, etc.^19^

In this study, it was aimed to obtain new water-soluble polymer–drug conjugates by combining antineoplastic drug active ingredients with copolymers showing many different biological activities due to their functional properties. For this purpose, due to the high side effects of the drugs gemcitabine and methotrexate, which are widely used in the clinic, experiments were carried out to reduce their toxicity and increase their anticancer potential by binding the drugs to the copolymer and to develop candidate molecules against the increasing drug resistance of microorganisms by examining the antimicrobial effects of copolymers and conjugates.

## Materials and Methods

### Materials

Analytical grade chemicals and antimicrobial cytotoxicity test agents were used as follows: MA (by recrystallization in anhydrous benzene), methyl ethyl ketone, and the radical initiator reagent benzoyl peroxide (BPO) were supplied by Merck. Petroleum ether, ethyl acetate, and vinyl acetate (VA) were obtained from Sigma-Aldrich. Dimethylformamide solvent (DMF) and triethylamine (Et_3_N) were purchased from Merck (Germany). DMEM (Dulbecco′s modified Eagle′s medium), Fetal Bovine Serum (FBS), and penicillin–streptomycin were obtained from Gibco (NewYork). MTT (3-(4,5-dimethylthiazol-2-yl)-2,5-diphenyltetrazolium bromide) was supplied by Thermo Fisher (USA).

### Synthesis and Purification of the Copolymer

Maleic anhydride-vinyl acetate copolymer (MAVA) synthesized and purified in our previous study was used as a carrier for drug agents.^20^

### Synthesis and Purification of Conjugates

Taking the conjugation method in another previous study as a reference, MAVA-methotrexate (MAVA–MTX) and MAVA–gemcitabine (MAVA–GEM) conjugates were prepared by a 1:1 mol ratio of pure MAVA copolymer with MTX and GEM antitumor agent in DMF under the catalysis of TEA at 50 °C for 2 h and 70 °C for 48 h for a total of 50 h. The conjugate was finally removed from the organic medium by precipitation with excess cold ethyl alcohol at −20 °C for 1 h. Furthermore, the conjugate was purified by drying in a vacuum oven at about 50 °C for 24 h.^21^

### Structural Characterization of the Copolymer and Conjugates

MAVA copolymer, MAVA–MTX, and MAVA–GEM conjugates were prepared as KBr pellets (2 mg sample, 100 mg KBr) and spectra were recorded with a Fourier transform infrared (FTIR) spectrophotometer (MATTSON 1000 Unicam, USA) at 400–4000 cm^–1^ with 4 cm^–1^ intervals. Nuclear magnetic resonance (^1^H NMR) analysis was performed at 400 MHz (Bruker Avance III, Karlsruhe, Germany) by preparing 6 mg samples in 0.8 mL of dimethyl sulfoxide (DMSO) (Technology Research and Application Center, Erciyes University, Kayseri, Turkey).

### Anticancer Activity

The anticancer activities of MAVA–MTX and MAVA–GEM conjugates, methotrexate, and gemcitabine at 6 different concentrations were determined using the MTT assay on MCF-7 and L929 cell lines. Cells were grown in DMEM (Dulbecco’s modified Eagle’s medium) medium and 10% FBS + 1% penicillin–streptomycin medium in an oven at 37 °C with 5% CO_2_. For the MTT cell metabolic activity assay, 96-well culture plates were prepared with 100 μL (1 × 10^4^ cells/well) per well. Six different concentrations of MAVA–MTX and MAVA–GEM conjugates (500–15.62 μg/mL) were added to the wells and incubated for 24 h. MTT (Cell proliferation kit, Roche) solution was prepared and 10 μL was added to each well and incubated at 37 °C for 4 h. The medium in the wells was removed and 100 μL of DMSO was added. Optical density was measured with an ELISA device at 570 nm wavelength. Results were calculated as % viability for MCF-7 and L929 using positive and negative control values using the following formula^22^
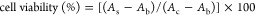
*A*_s_: absorbance sample, *A*_b_: absorbance blank, *A*_c_: absorbance viable cell (control).

### Preparation of Test Microorganisms

Pathogenic strains of *Staphylococcus aureus* (ATCC 25923), Methicillin-Resistant *S. aureus* (MRSA) (ATCC 43300), *Enterococcus faecalis* (ATCC 29212), *Pseudomonas aeruginosa* (ATCC 27853), *Escherichia coli* (ATCC 25922), and *Candida albicans* (ATCC 10231) were used for the tests. Bacterial strains were inoculated in Brain Heart Infusion Broth and incubated at 37 °C, and fungal strains were inoculated in Sabouraud Dextrose Broth and incubated at 25 °C for 24 h. Bacterial and yeast solutions from these cultures were adjusted to the 0.5 standard on a McFarland apparatus.^23^

### Antimicrobial Activity

#### Disk Diffusion Test

The disk diffusion method was applied to determine the antimicrobial activity of the copolymers and conjugates. Bacterial and yeast cultures (adjusted to 0.5 McFarland standard) were inoculated on Muller–Hinton agar plates using the smear inoculation method. The plates were dried for 15 min and then used for susceptibility testing. The copolymer and conjugates were dissolved in DMSO. Dilutions were made in sterile tubes containing 1 mL of DMSO. The solutions were impregnated with 25 μL on sterile 6 mm blank discs (OXOID blanc disc). Antibiotic discs were used as positive controls. Positive controls were placed in the center of the Petri dish with 20 mm between them and the other copolymer and conjugate discs. Bacteria inoculated plates were incubated at 37 °C for 24 h, and yeast-inoculated plates were incubated at 25 °C for 48 h. At the end of the time, the zones of inhibition formed around the discs on the medium were evaluated in mm.^24^ The experiments were repeated 3 times, and the results were analyzed statistically.

#### Minimum Inhibitory Concentration

To investigate the antimicrobial activity of the copolymer and conjugates, the Minimal Inhibitory Concentrations (MICs) were determined following the microdilution procedures described by the European Committee for Antimicrobial Susceptibility Testing (EUCAST).^24^ Suspensions dissolved in DMSO were added to U-bottom 96-well plates in 2-fold serial dilutions. Bacterial and yeast cultures adjusted to 0.5 McFarland were then transferred to each well, along with the media. After incubation at 37 °C for 24 h, the lowest concentration of the copolymer and conjugates that inhibited visible growth was recorded as MIC. All MIC values were obtained in triplicate. In addition, the degree of agreement in all experiments was 3/3 and the standard deviation (±SD) was zero.^25^

### Statistical Analysis

Statistical evaluation of the results was performed with the SPSS (Statistical Packages of Social Sciences version 29.0 for Windows, Mann–Whitney *U* Test) program. A value of “*p* < 0.05” was considered statistically significant. Differences between more than two independent groups were evaluated by the Kruskal–Wallis test.

## Results

### Structural Characterization of the Copolymer

Structural characterization of the MAVA copolymer was performed by FTIR spectrophotometry and nuclear magnetic resonance spectrometry (^1^H NMR).

### FTIR Analysis of the MAVA Copolymer

**Figure 1:**
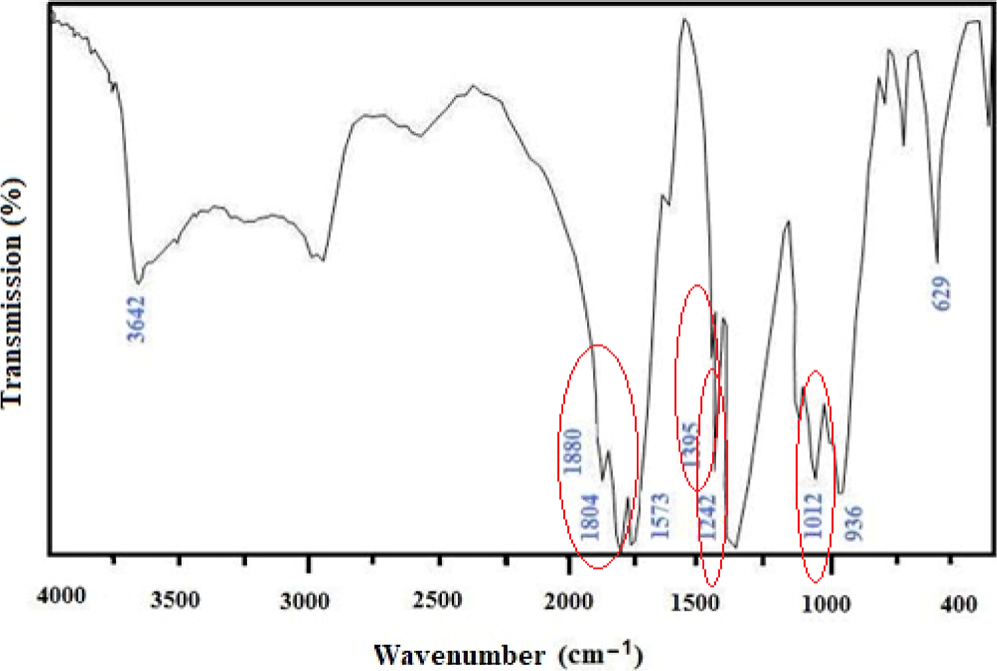
FTIR spectrum of the MAVA copolymer.

As can be seen in the spectrum in Figure 1, the characteristic peaks at 1880 and 1804 cm^–1^ of MA, which is the regular unit in the copolymer structure, are the stress vibrations of the carbonyl (C=O) group of symmetric and asymmetric anhydride, respectively.^26^ The characteristic peaks at 1242 cm^–1^ and 1012 cm^–1^ are the stretching vibration of the C–O bond and the peak at 1395 cm^–1^ is the stretching vibration of the methyl (CH_3_) group in VA.^27,28^

### ^1^H NMR Analysis of the MAVA Copolymer

**Figure 2:**
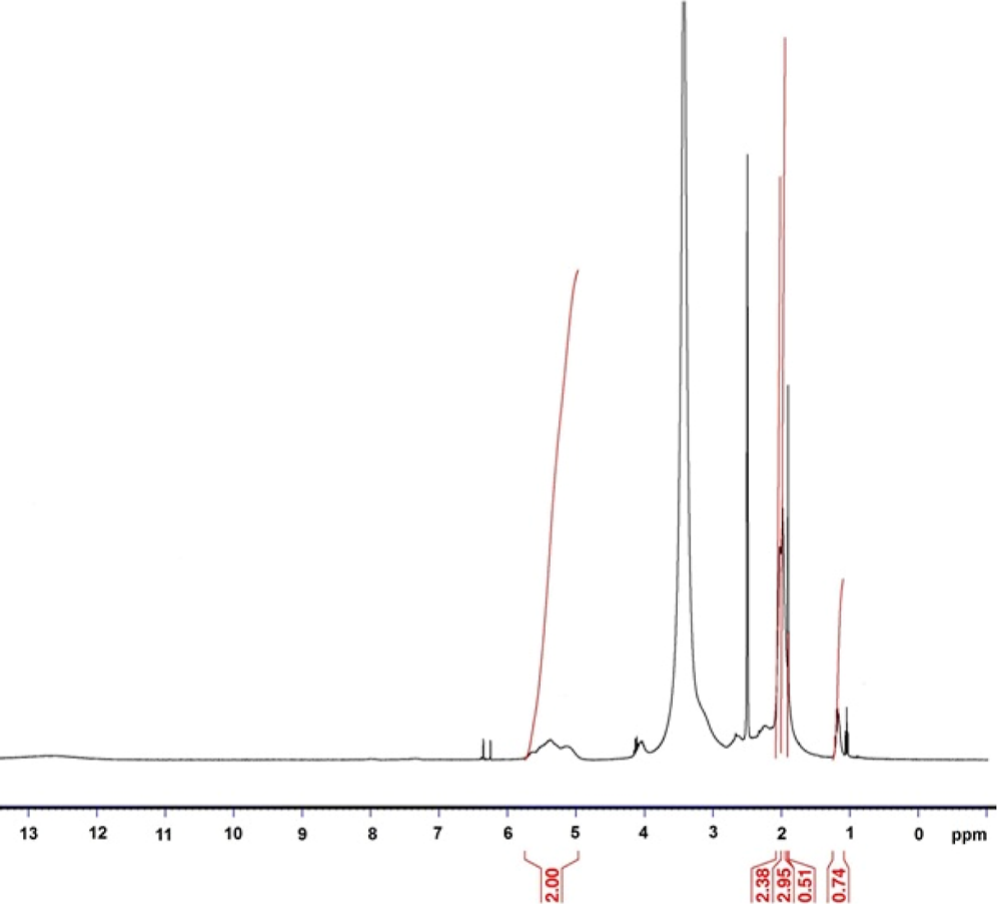
^1^H NMR spectrum of the MAVA copolymer.

The ^1^H NMR results support the FTIR results obtained for the MAVA copolymer. The MAVA spectrum (Figure 2) exhibits characteristic peaks appearing as two protons on the MA groups (−CH–CH−) at 5.4 ppm, and three methyl (−CH_3_) protons at 2 ppm,^29,30^ a chemical shift of the –CH_2_ protons on VA at ∼2 ppm, and a multiplet peak at ∼1.1 ppm for –CH adjacent to oxygen.^28,31^

### Structural Characterization of Copolymer–Drug Pairs

The spectra obtained by FTIR spectroscopy were used only to predict whether the anhydride ring of MA was opened by the active ingredient. For this reason, NMR analysis was also required since FTIR did not provide sufficient structure elucidation and only gave an idea of whether this ring was opened or not.^32^

### FTIR Analysis of MAVA–MTX Conjugate

**Figure 3:**
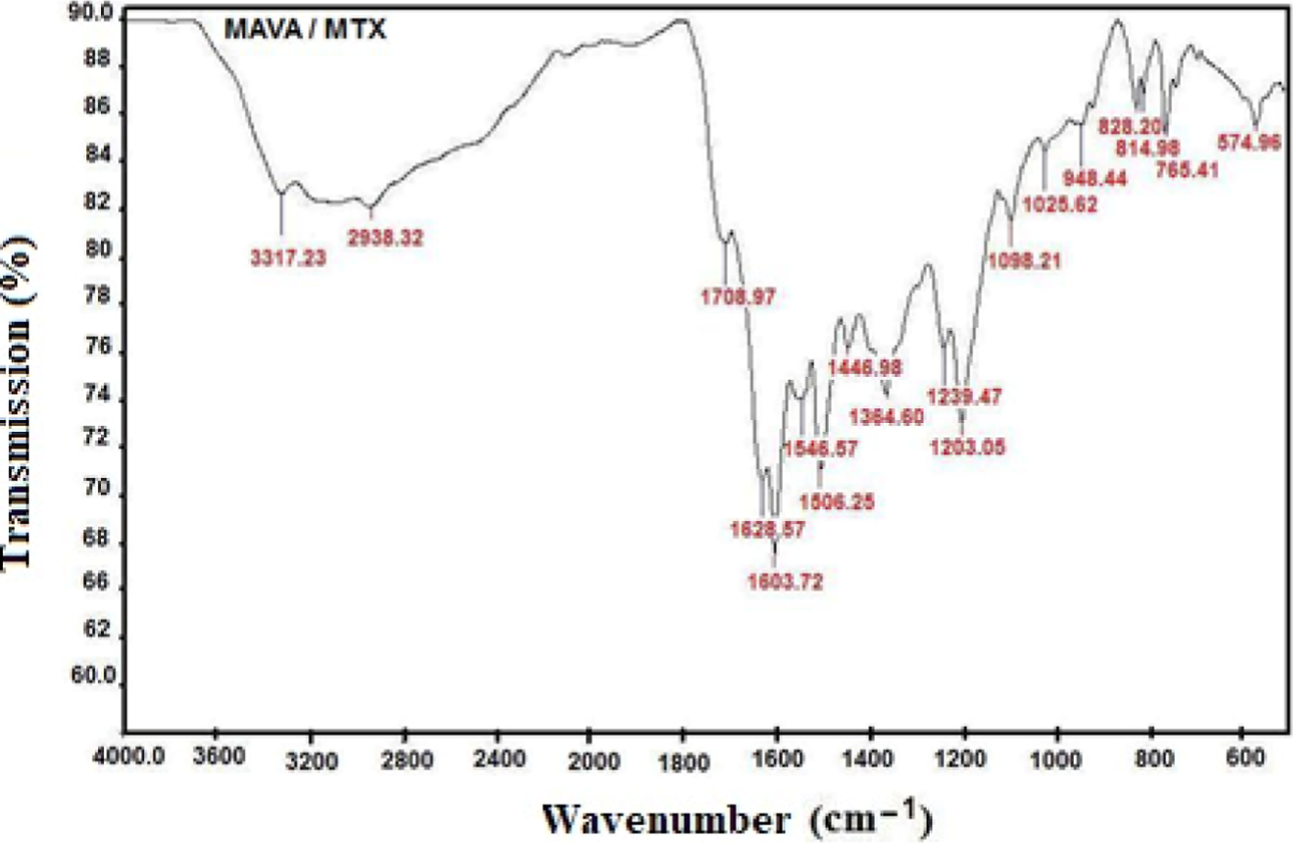
FTIR spectrum of the MAVA–MTX conjugate.

The characteristic peaks at 1880 and 1804 cm^–1^, which are the symmetric and asymmetric carbonyl (C=O) group stretching vibrations of MA, the most characteristic unit seen in the structure of the copolymer, completely disappeared.^33^ This is caused by the opening of the anhydride ring in the structure with the active ingredient MTX. The characteristic peak at 1239.47 cm^–1^ in the spectrum in Figure 3 is the stretching vibration of the C–O bond of the MAVA copolymer.^28^ Other peaks observed in the MAVA/MTX spectrum; 3317.23 cm^–1^, 1628.57 cm^–1^, 1603.72 cm^–1^, 1546.57 cm^–1^, 1506.25 cm^–1^, 1446.98 cm^–1^, 1364.60 cm^–1^, 1203.05 cm^–1^, 1098.21 cm^–1^, 948.44 cm^–1^, 828.20 cm^–1^, 814.98 cm^–1^, 765.41 cm^–1^, and 574.96 cm^–1^ are due to the characteristic functional group peaks of the MTX molecule. The other 3 peaks in the spectrum are 2938.32 cm^–1^, 1708.97 cm^–1^, and 1025.62 cm^–1^, respectively. The 2938.32 cm^–1^ peak at high chemical shift frequency is due to the C–H stretching in the structure.^26,34^ The amide (−N–C=O bond) or carboxylic acid (−COOH bond) peak, which should normally appear around 1695, was recorded around 1708.97 cm^–1^ with a very slight shift.^35^ The appearance of amide/carboxylic acid peaks in the structure is the most important evidence that the MTX drug is bound to the polymer and the 1025.62 cm^–1^ peak is due to the C–O–C stretching vibration of the MA ring.^36,37^

### FTIR Analysis of the MAVA–GEM Conjugate

**Figure 4:**
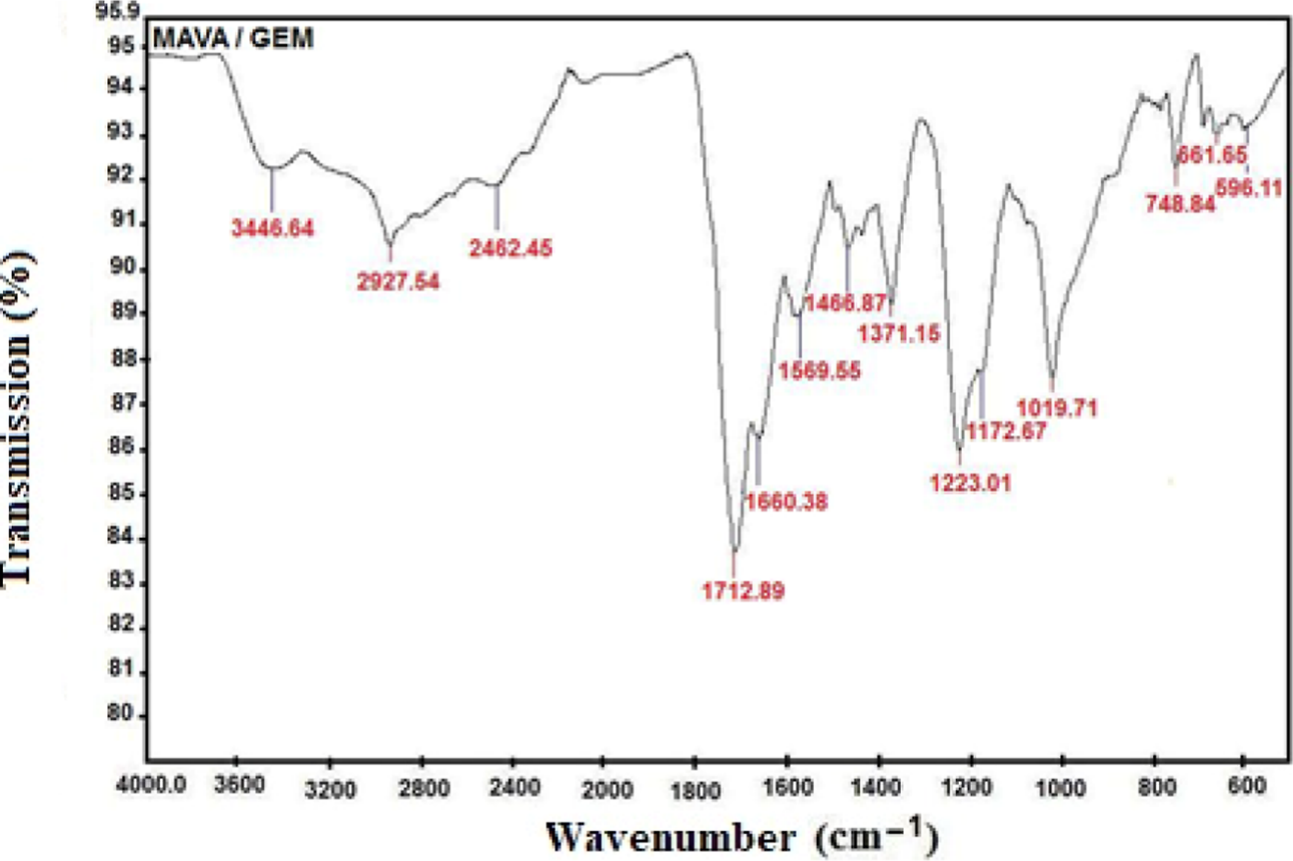
FTIR spectrum of the MAVA–GEM conjugate.

The characteristic peaks at 1880 and 1804 cm^–1^, which are the stress vibrations of the symmetric and asymmetric anhydride carbonyl (C=O) group of MA, the most characteristic unit seen in the structure of the copolymer in Figure 4, have completely disappeared.^33,35^ This change is entirely a result of the opening of the anhydride ring in the copolymer structure by the nucleophilic attacking drug active ingredient GEM. The other peaks observed in the MAVA–GEM spectrum, 2927.54 cm^–1^, 1466.87 cm^–1^, 1371.15 cm^–1^, 1019.71 cm^–1^, and 748.84 cm^–1^ are due to the characteristic functional groups of the GEM molecule. Other peaks recorded in the spectrum are as follows; C=O stretching vibration of the copolymer at 1712.89 cm^–1^^38^ at 1660.38 cm^–1^,^36^ N–H bond of the amide group at 1569.55 cm^–1^^39^ peak belonging to the CH_2_ group in the structure, and 596.11 cm^–1^^35^ is the peak due to C–C stretching. Also, 3446.64 cm^–1^^40^ are the vibrations of the OH group.

### ^1^H NMR Analysis

#### ^1^H NMR Analysis of the MAVA–MTX Conjugate

**Figure 5:**
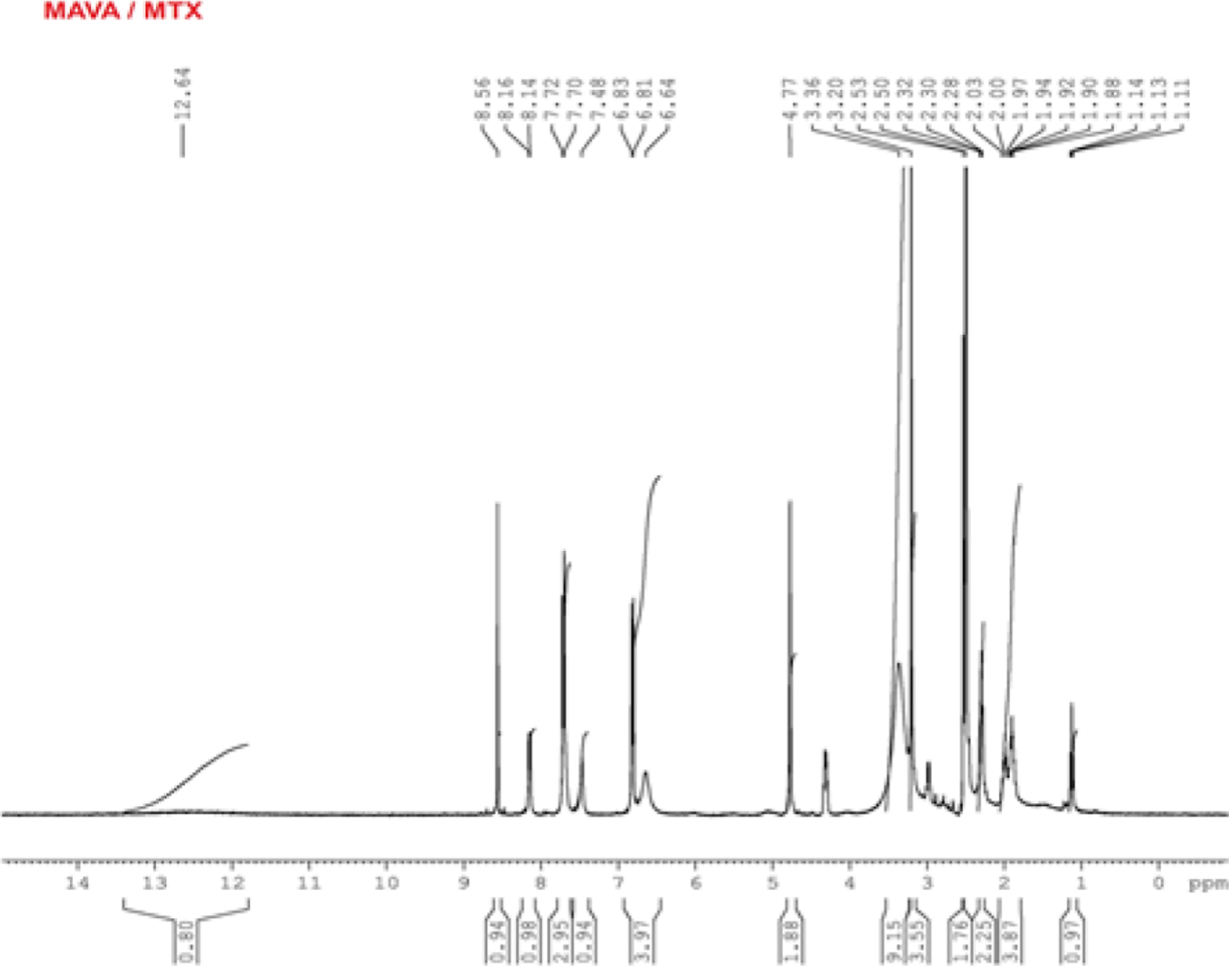
^1^H NMR spectrum of the MAVA–MTX conjugate.

As seen in Figure 5, the chemical shift value of the 2 protons of MA in the MAVA copolymer is around 5.4 ppm and shifted to 4.77 ppm in this spectrum.^28^ The methyl (−CH_3_) protons of VA were observed at 1.94 and 1.97 ppm in the spectrum, very close to 2 ppm.^29,30^ The methylene –CH_2_ peaks of the copolymer belonging to VA appeared around 2 ppm right next to this peak. Again, the oxygen-bound –CH peak associated with the copolymer was observed at 1.11, 1.13, and 1.14 ppm as a multiplet around 1.1 ppm in the spectrum.^31^ In this spectrum, the most important evidence for the binding of the active substance MTX to the MAVA copolymer is that the characteristic aromatic functional group peaks of the active substance, 6.64, 6.81, 6.83, 7.48, 7.70, 7.72, 8.14, 8.16, and 8.56 ppm, were recorded.^41,42^ Another evidence of binding is that the –NH_2_ group peaks, which usually appear around 10.55 ppm in the spectrum due to unbound MTX, were not observed. In addition, characteristic carboxyl (−COOH) group protons on MTX moieties formed a peak at 12.64 ppm.^43^

Considering the results, it can be said that the active substance MTX binds to the MAVA copolymer via a nucleophilic reaction with a highly successful amidation mechanism.

#### ^1^H NMR Analysis of the MAVA–GEM Conjugate

**Figure 6:**
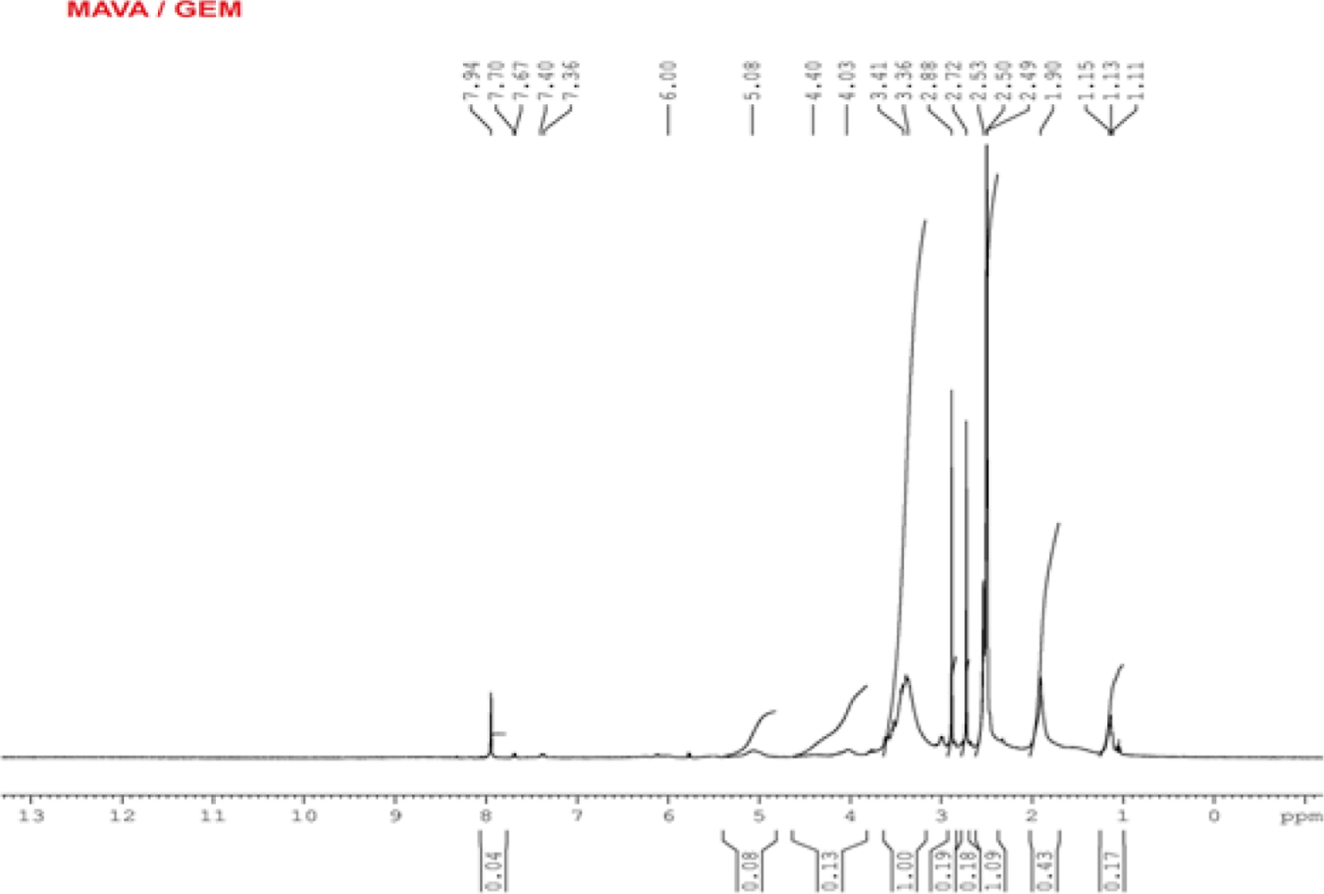
^1^H NMR spectrum of the MAVA–GEM conjugate.

As seen in Figure 6, the chemical shift value of 2 protons belonging to MA in the MAVA copolymer is around 5.4 ppm and shifted to 5.08 ppm in this spectrum.^28^ Again, the oxygen-bound –CH peak associated with the MAVA copolymer was observed at 1.11, 1.13, and 1.15 ppm as a multiplet around 1.1 ppm in the spectrum.^31^ Methyl (−CH_3_) protons should have been observed around 2 ppm in the spectrum but were masked by the group peaks of the active substance GEM. Methylene –CH_2_ peaks were observed at 1.90 ppm in the spectrum, while they are normally observed at around 2 ppm. The –CH protons of MA appeared at 2.53, 2.72, and 2.88 ppm. Amide, –NH (−CONH−) protons were also detected at 7.36, 7.40, 7.67, 7.70, and 7.94 ppm, confirming that GEM bound to the MAVA copolymer backbone by an amidation reaction.^44^ In addition, additional evidence for binding is that the –NH_2_ group peaks that usually appear around 10.55 ppm in the spectrum due to unbound GEM are not observed. In this spectrum, the most important evidence of the binding of the active substance to the MAVA copolymer is the observation of the signal belonging to the –NH(−CONH−) amide group, although weak.

Considering the results, it can be said that the GEM active substance binds to MAVA copolymer by amidization mechanism.

#### Solubility and Physical Properties of Copolymer–Drug Conjugates

Considering the structural characterization of the synthesized conjugates confirmed by FTIR and ^1^H NMR spectroscopic methods, it can be said that the MAVA copolymer is generally partially soluble in water and completely soluble in organic solvents. In particular, as the rate of binding of drugs with low or no water solubility to the copolymer increases, the water solubility increases accordingly. The following can be said for the solubility and some physical properties of the synthesized copolymers and conjugates: MAVA is in the form of white powder and its aqueous solution is colorless, MAVA–MTX conjugate is in the form of powder, dark yellow, and although it dissolves very well in water, the color of its aqueous solution is yellow. The MAVA–GEM conjugate is powdery, dark brown, and dissolves fairly well in water, but its aqueous solution is brown.

#### Reaction Mechanism for the MAVA–MTX and MAVA–GEM Conjugates

**Figure 7:**
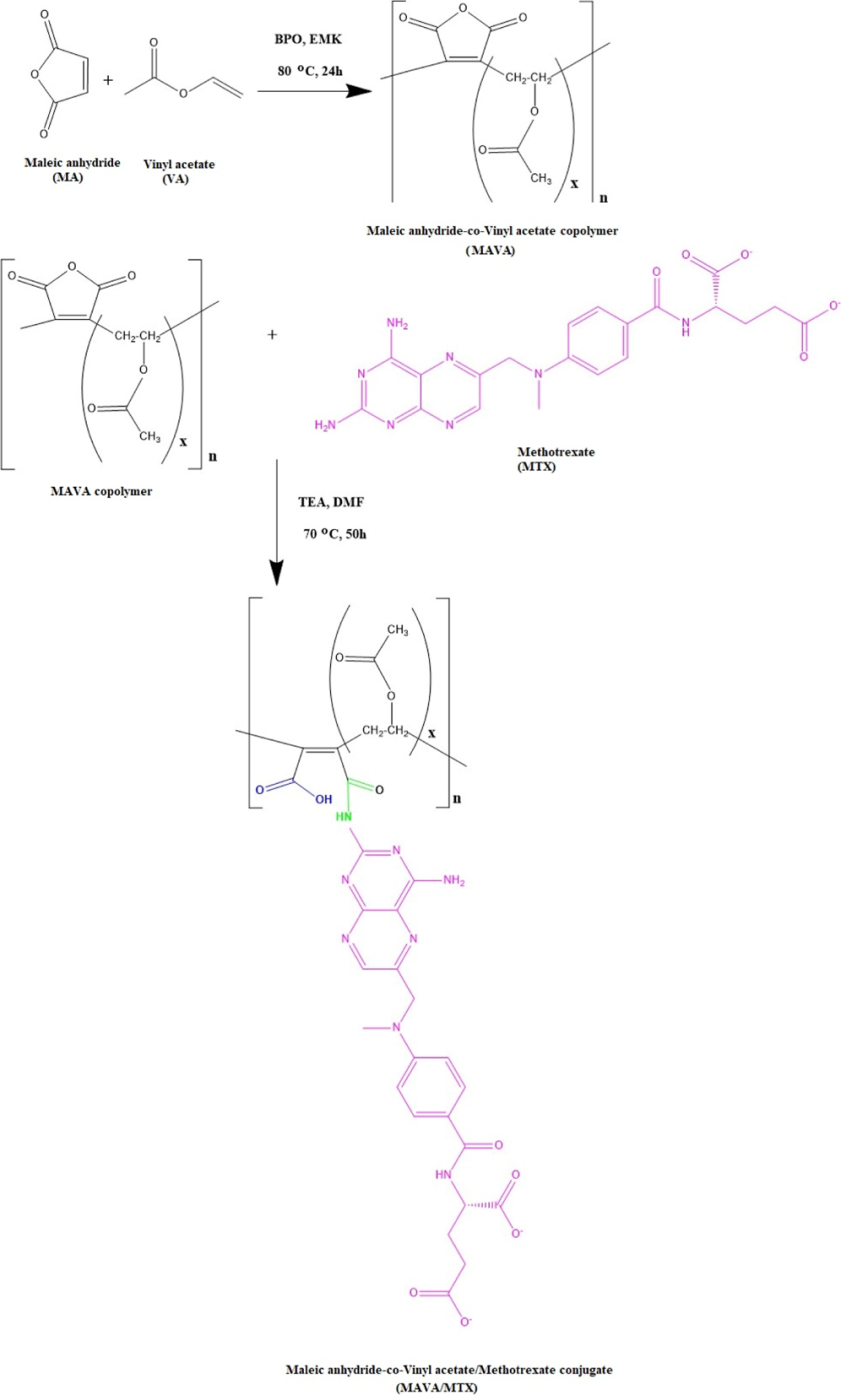
Conjugation reaction of the MAVA copolymer with the MTX anticancer agent.

**Figure 8:**
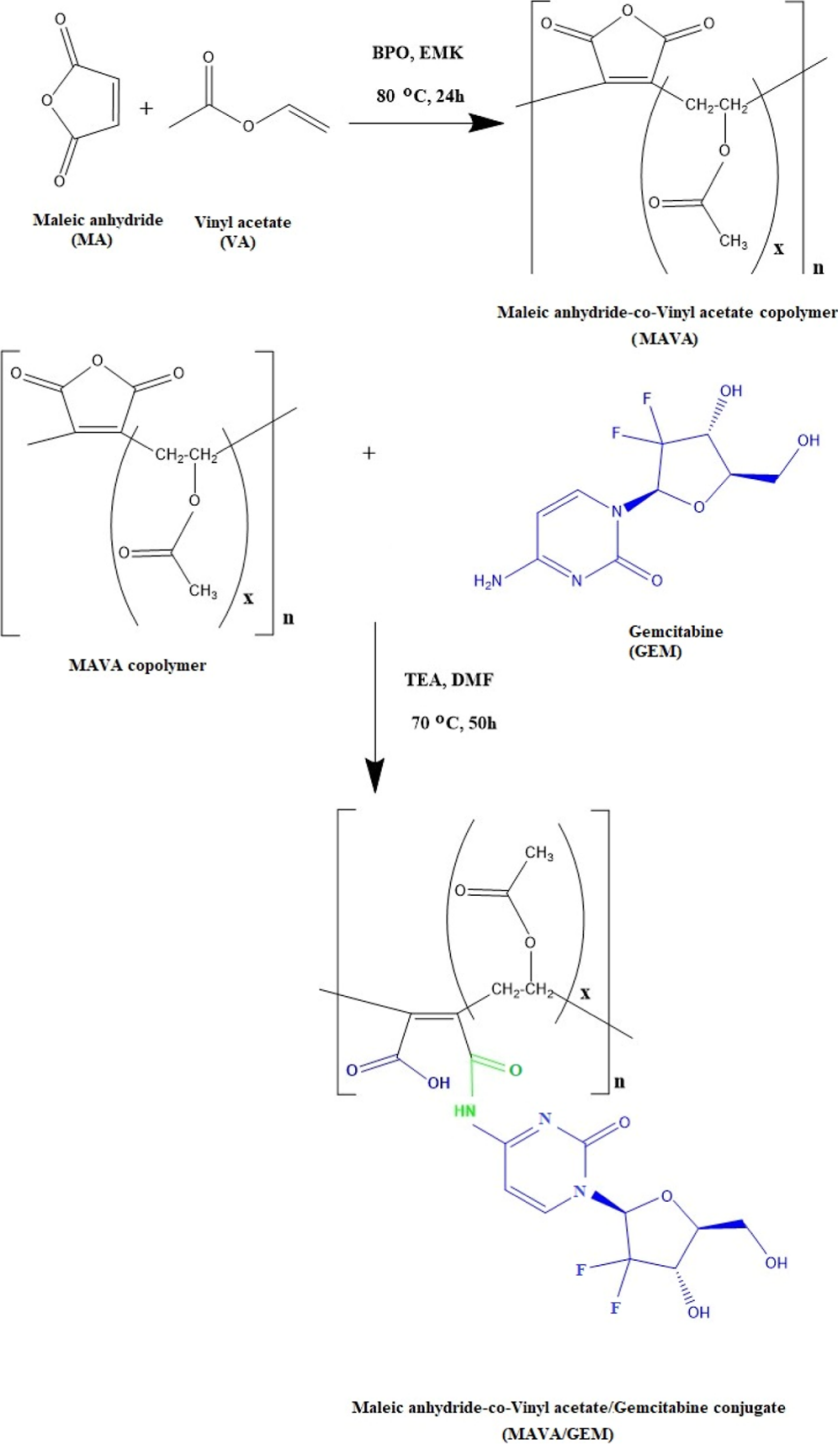
Conjugation reaction of the MAVA copolymer with the GEM anticancer agent.

Results from FTIR and ^1^H NMR spectroscopic methods revealed that the structure of the reaction product was compatible with free radical polymerization of vinyl-based monomers for the MAVA copolymer and that both MTX and GEM molecules were successfully combined into the copolymer backbone by a ring-opening reaction (Figures 7 and 8, respectively).

### Anticancer Activity

The anticancer activities of MAVA–GEM and MAVA–MTX conjugates and GEM and MTX drugs at 6 different concentrations were determined using MTT assay on MCF-7 and L929 cell lines (*p* < 0.05).

Averages of the 6 wells were taken for positive and negative controls and each different drug and copolymer–drug concentration. The % cell viability was calculated using the relevant formula, and the graph was obtained.

**Figure 9:**
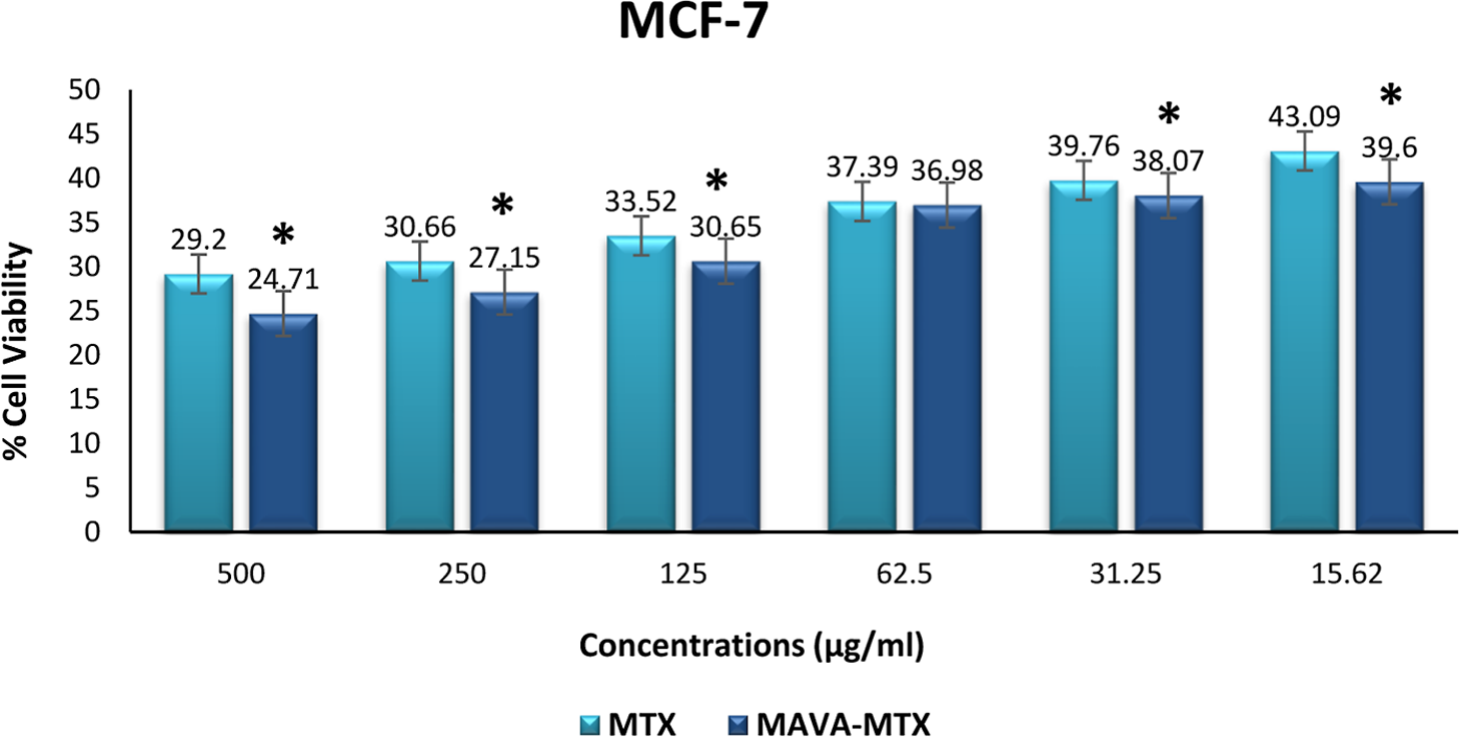
Cell viability of different concentrations of the MAVA–MTX conjugate and methotrexate on the MCF-7 cell line (*p < 0.05).

A statistically significant difference was found when cell viability at concentrations of 500 μg/mL, 250 μg/mL, 125 μg/mL, 31.25 μg/mL, and 15.62 μg/mL used for Methotrexate was compared with MAVA–MTX conjugate concentration data (*p* < 0.05). For the 62.5 μg/mL concentration, no significant difference was found between the MAVA–MTX conjugate and MTX drug (*p* > 0.05). Furthermore, the results of the Kruskal–Wallis test showed that there was a significant difference between all groups with varying concentrations (*p* < 0.001). The statistical significance level was accepted as *p* < 0.05 (Figure 9). When comparing methotrexate and MAVA–MTX from the highest to the lowest concentration, the rate of cancer cell killing of the MAVA–MTX conjugate was always higher than that of the drug alone, except for one concentration.

**Figure 10:**
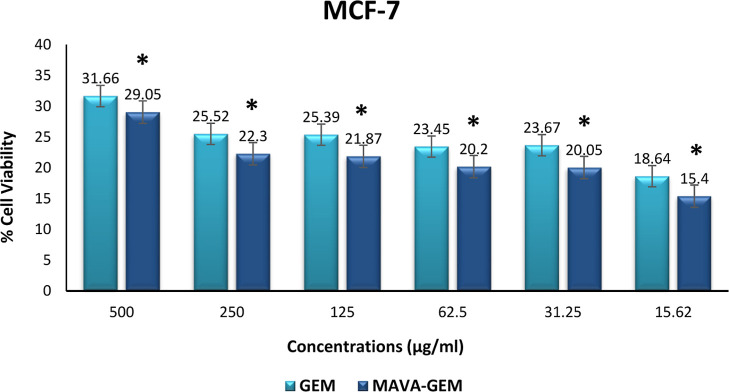
Cell viability of different concentrations of the MAVA–GEM conjugate and gemcitabine on the MCF-7 cell line (*p < 0.05).

There was a statistically significant difference in MCF-7 cell viability between the MAVA–GEM conjugate and gemcitabine at all concentrations (*p* < 0.05). Furthermore, Kruskal–Wallis test results showed that there was a significant difference between all groups including variable concentrations (*p* < 0.001). The statistical significance level was accepted as *p* < 0.05 (Figure 10). When comparing gemcitabine and MAVA–GEM from the highest to the lowest concentration, the rate at which the MAVA–GEM conjugate killed cancer cells was always higher than that of gemcitabine alone.

**Figure 11:**
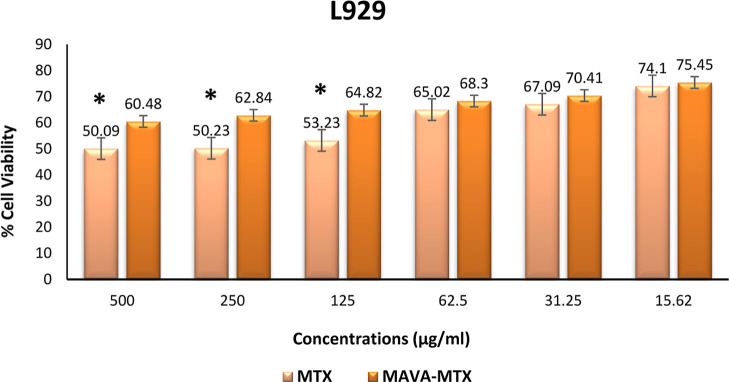
Cell viability of different concentrations of the MAVA–MTX conjugate and methotrexate on the L929 cell line (*p < 0.05).

There was a significant difference between the cell viability of 500 μg/mL, 250 μg/mL, and 125 μg/mL concentrations used for the Methotrexate and the cell viability of MAVA–MTX conjugate used at the same concentrations (*p* < 0.05). For 62.5 μg/mL, 31.25 μg/mL, and 15.62 μg/mL concentrations, no significant difference was found between the MAVA–MTX conjugate and Methotrexate (*p* > 0.05). The Kruskal–Wallis test results showed a significant difference between all groups including variable concentrations (*p* = 0.01). The statistical significance level was accepted as *p* < 0.05. The copolymer–drug conjugate was found to have a viability at all concentrations higher than that of the drug alone (Figure 11).

**Figure 12:**
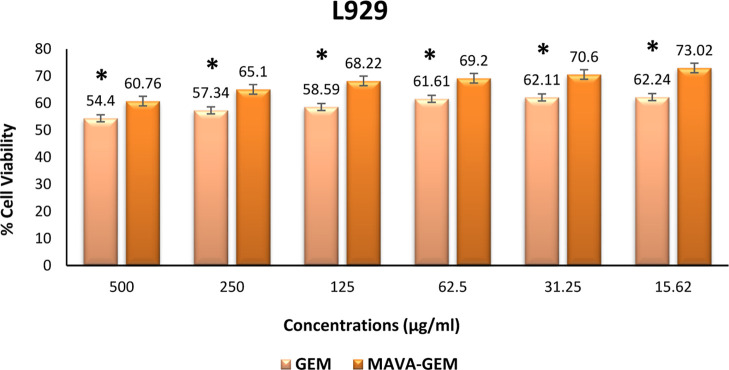
Cell viability of different concentrations of the MAVA–GEM conjugate and gemcitabine on the L929 cell line (*p < 0.05).

In terms of cell viability, a statistically significant difference was determined between all concentrations of the MAVA–GEM conjugate and gemcitabine applied to L929 cells (*p* < 0.05). Furthermore, Kruskal–Wallis test results showed that there was a significant difference between all groups including variable concentrations (*p* = 0.002). The statistical significance level was accepted as *p* < 0.05. The copolymer–drug conjugate was found to have a viability at all concentrations higher than that of the drug alone (Figure 12).

### Antimicrobial Activity

*S. aureus*, *E. faecalis*, Meticillin-Resistant *S. aureus* as Gram-positive bacteria; *E. coli* and *P. aeruginosa* as Gram-negative bacteria; *C. albicans* strains as fungi were used to investigate the antimicrobial activity of copolymers and conjugates. The evaluation was performed by disc diffusion and MIC tests.

**Figure 13:**
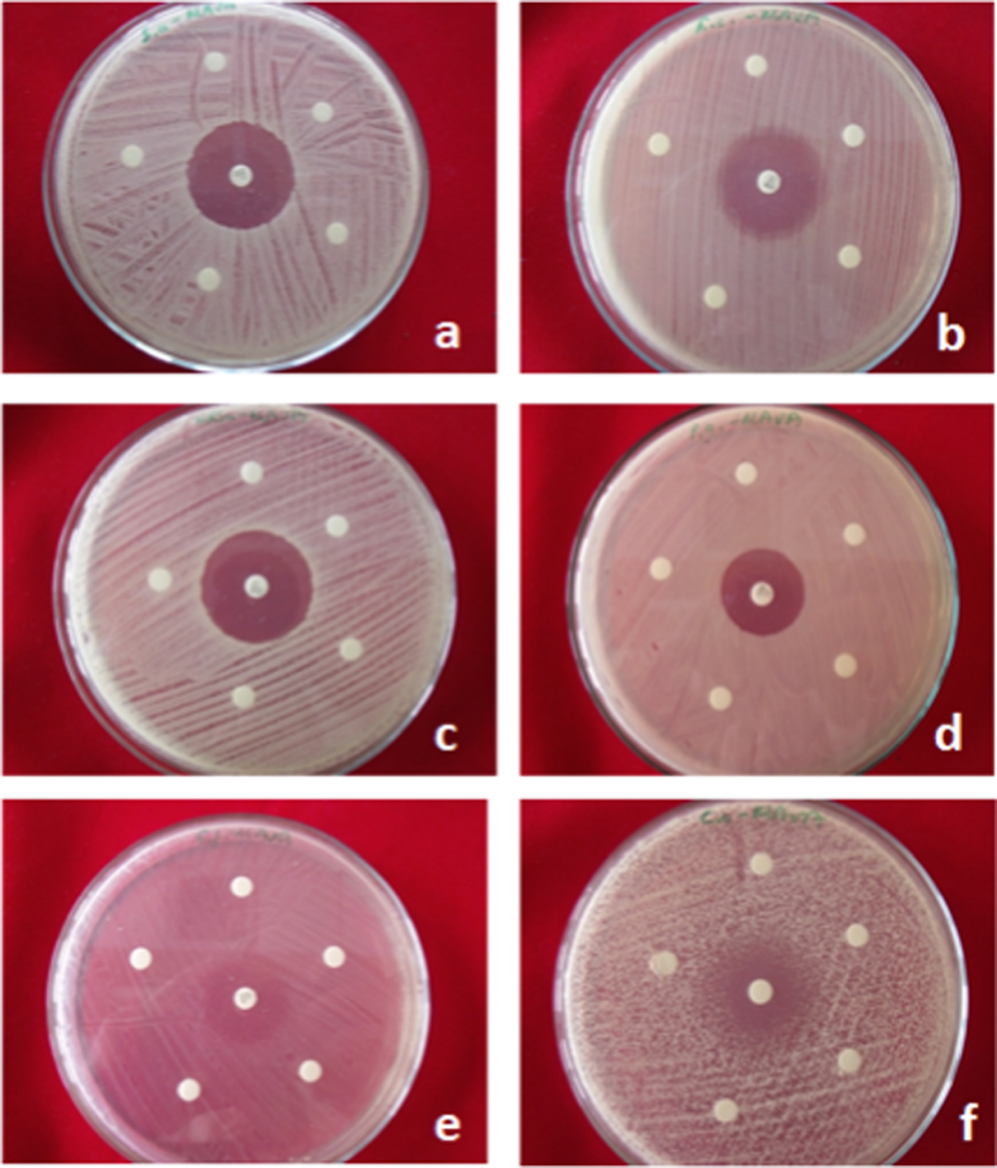
(a) *S. aureus*, (b) *E. coli*, (c) MRSA, (d) *P. aeruginosa*, (e) *E. faecalis*, (f) *C. albicans*, disc diffusion results of copolymers and conjugates.

The disc diffusion results determined that the tested MAVA copolymer did not form a visible zone of inhibition on the microorganisms (Figure 13).

Results were compared with reference sources and MIC values of standard antibiotics. The reference source is given as [effective (MIC < 100 μg/mL), moderate (100 < MIC ≤ 625 μg/mL), and weak (MIC > 625 μg/mL)]. MIC values of copolymers, conjugates, and antibiotics are given in Table 1.

**Table 1:** MIC Results of MAVA, MAVA–MTX, and MAVA–GEM (μg/mL)

microorganisms (bacteria and yeast)	MIC (μg/mL) MAVA	MIC (μg/mL) MAVA–MTX	MIC (μg/mL) MAVA–GEM	MIC (mg/L) antibiotics	antibiotics
*E. coli*	200	200	100	64	Nitrofurantoin
*P. aeruginosa*	100	200	200	16	Amikacin
*S. aureus*	200	100	100	16	Amikacin
*E. faecalis*	100	200	100	64	Nitrofurantoin
MRSA	100	200	200	16	Amikacin
*C. albicans*	200	100	100	0.25	Fluconazole

The copolymers and conjugates, found to be moderately effective according to reference sources, were not found to be antimicrobially effective when compared with the MIC values of reference antibiotics.

## Discussion

According to clinically approved products in the field of drug development, the main aim is to localize drugs to where they need to go in the body and reduce side effects. Particularly, polymer-based drug carrier systems are generally used in biomedical applications to deliver therapeutic agents to target cells. Benefits such as decreasing drug toxicity, increasing the water solubility of the drug, bioavailability, and biocompatibility enable it to improve many properties of existing drugs as a next-generation drug delivery system. In light of this information, polymer-based drug conjugates designed with methotrexate and gemcitabine have very promising potential for cancer research.

This study aims to design MAVA–MTX and MAVA–GEM conjugate forms of MTX and gemcitabine (GEM), which are currently used effectively in the clinic for the treatment of cancer, to reduce the existing toxicity, side effects, and similar undesirable effects using a poly(MA-*co*-vinyl acetate) copolymer. As is well-known, the use of high-dose methotrexate (HDMTX) and gemcitabine is associated with many other undesirable side effects, including toxicity.^45^ Alternative ways of dealing with this toxicity may be developed, such as preparing the conjugate form and using it at low doses. Drug design studies generally aim to improve solubility, increase bioavailability, and reduce toxicity. As a result, from a toxicity perspective, MAVA–MTX had a less toxic effect on healthy L929 cells than MTX.^46^

On the other hand, when MAVA–MTX and MAVA–GEM were compared, it was concluded that the MAVA–GEM conjugate had less toxic effects, i.e., it caused less damage to healthy cells. When the results were examined in terms of anticancer potential, it was found that the MAVA–MTX conjugate killed MCF-7 cells more than free MTX at all concentrations. When the two conjugates are compared, the MAVA–GEM conjugate has a higher rate of killing MCF-7 cells.

Many studies in the literature highlight the advantages of a drug’s conjugate form. An example of such studies is testing of the synergistic effect of the pH-sensitive pentaerythritol-grafted MA copolymer synthesized by Rahmani et al. with doxorubicin on MDA-MB-231 breast cancer cells. According to the results, the polymer–drug conjugate was found to be effective at lower doses.^47^

In our previous study, we investigated the cytotoxic effects of the conjugate formed by the MAVA copolymer used as a carrier and the drug known as cytarabine on MCF-7 cells. The results obtained showed that the anticancer activity was not increased, but the conjugate produced almost no toxic effect on healthy cells compared to the drug.^48^

In a more comprehensive study, Zarrinzadeh et al. investigated the anticancer and antimicrobial properties of a conjugate they synthesized from euphralin and a MA copolymer and showed that it was effective against MDA-MB-231 breast cancer cells. More strikingly, this MA-containing derivative was found to be specifically effective only against Gram-positive bacteria.^49^

## Conclusions

In this study, the anticancer activity of the MAVA–MTX copolymer–drug conjugate on MCF-7 cells was statistically higher than the single drug methotrexate. Moreover, the toxicity of MAVA–MTX on healthy L929 cells was also statistically lower than for MTX. Our other synthesized MAVA–GEM copolymer–drug conjugate showed statistically significantly higher anticancer activity than single gemcitabine at all concentrations on MCF-7 cells. At the same time, the toxicity of MAVA–GEM to healthy L929 cells was also statistically lower than that of gemcitabine. Based on these results, it can be concluded that the newly synthesized MAVA–MTX and MAVA–GEM polymer–drug conjugates are potential drug carrier candidates for breast cancer treatment.

In addition, although the carrier chosen as the copolymer is common to both conjugates, there are differences in the structure and efficiency between the two conjugates obtained. According to the cell viability test, it was strikingly observed that MAVA–GEM was much more effective than MAVA–MTX. This situation is due to the chemical structure of the conjugates, but from a structure–activity relationship (SAR) point of view, it is known that in crowded by-volume groups and high molecular mass structures such as MAVA–MTX, a decrease in activity usually occurs with the appearance of steric hindrance. It is well-known that macromolecular crowding affects enzymatic activity, protein folding, small molecule binding, nucleic acid interaction, protein–protein interactions, etc. Therefore, this result is significant, as the MTX molecule is larger than the GEM molecule in both molecular mass and volume. The fact that the conjugate forms have a greater anticancer effect than the free drugs they contain can be explained as a result of the increase in polarity with the appearance of carboxyl and amide groups and the consequent increase in solubility.

In the direction of our main goal, in addition to lower toxicity, the enhanced anticancer effects of antineoplastic drugs on cancer cells are the main target, and we have successfully obtained good water-soluble copolymer–drug couples with lower cytotoxicity than the free drug and also higher anticancer activity than the copolymer and the single drug, at all doses toward selected cancer cells. In conclusion, both polymer–drug conjugates are effective pretreatment agents for experimental animal studies and clinical trials due to their reduced side effects and enhanced anticancer activity.
